# Evogliptin, a DPP-4 inhibitor, prevents diabetic cardiomyopathy by alleviating cardiac lipotoxicity in db/db mice

**DOI:** 10.1038/s12276-023-00958-6

**Published:** 2023-04-03

**Authors:** Trong Kha Pham, To Hoai T. Nguyen, Joo Mi Yi, Gwang Sil Kim, Hyeong Rok Yun, Hyoung Kyu Kim, Jong Chul Won

**Affiliations:** 1 Cardiovascular and Metabolic Disease Center, Smart Marine Therapeutic Center, Department of Physiology, College of Medicine, Inje University, Busan, South Korea; 2 Department of Health Sciences and Technology, Graduate School, Inje University, Busan, South Korea; 3 University of Science, Vietnam National University, Hanoi, Vietnam; 4 Department of Microbiology and Immunology, College of Medicine, Inje University, Busan, South Korea; 5 Division of Cardiology, Department of Internal Medicine, Sanggye Paik Hospital, Inje University, Seoul, South Korea; 6 Division of Endocrinology and Metabolism, Department of Internal Medicine, Sanggye Paik Hospital, Cardiovascular and Metabolic Disease Center, College of Medicine, Inje University, Seoul, South Korea

**Keywords:** Cardiomyopathies, Energy metabolism

## Abstract

Dipeptidyl peptidase-4 (DPP-4) inhibitors are glucose-lowering drugs for type 2 diabetes mellitus (T2DM). We investigated whether evogliptin® (EVO), a DPP-4 inhibitor, could protect against diabetic cardiomyopathy (DCM) and the underlying mechanisms. Eight-week-old diabetic and obese db/db mice were administered EVO (100 mg/kg/day) daily by oral gavage for 12 weeks. db/db control mice and C57BLKS/J as wild-type (WT) mice received equal amounts of the vehicle. In addition to the hypoglycemic effect, we examined the improvement in cardiac contraction/relaxation ability, cardiac fibrosis, and myocardial hypertrophy by EVO treatment. To identify the mechanisms underlying the improvement in diabetic cardiomyopathy by EVO treatment, its effect on lipotoxicity and the mitochondrial damage caused by lipid droplet accumulation in the myocardium were analyzed. EVO lowered the blood glucose and HbA1c levels and improved insulin sensitivity but did not affect the body weight or blood lipid profile. Cardiac systolic/diastolic function, hypertrophy, and fibrosis were improved in the EVO-treated group. EVO prevented cardiac lipotoxicity by reducing the accumulation of lipid droplets in the myocardium through suppression of CD36, ACSL1, FABP3, PPARgamma, and DGAT1 and enhancement of the phosphorylation of FOXO1, indicating its inhibition. The EVO-mediated improvement in mitochondrial function and reduction in damage were achieved through activation of PGC1a/NRF1/TFAM, which activates mitochondrial biogenesis. RNA-seq results for the whole heart confirmed that EVO treatment mainly affected the differentially expressed genes (DEGs) related to lipid metabolism. Collectively, these findings demonstrate that EVO improves cardiac function by reducing lipotoxicity and mitochondrial injury and provides a potential therapeutic option for DCM.

## Diabetes: A treatment for preserving heart health

A drug for managing glucose levels in type 2 diabetes can also preserve heart function by regulating fatty acid metabolism in cardiovascular tissues. Cardiomyopathy is a serious complication associated with diabetes, putting patients at risk of heart failure. There is evidence that diabetes drugs targeting the enzyme DPP-4 can improve heart health, and researchers led by Hyoung Kyu Kim and Jong Chul Won at Inje University, Busan and Seoul, South Korea, evaluated one such drug, evogliptin, in a mouse model of diabetes. They showed that in addition to controlling blood sugar, this drug also improved blood pressure and overall cardiac function. Evogliptin also reduced abnormal accumulation of fat and minimized the resulting disruption of normal metabolic function in heart muscle cells. These results highlight a new opportunity to manage diabetic cardiomyopathy.

## Introduction

Type 2 diabetes mellitus (T2DM), a common metabolic disease, accounts for over 90% of diabetes cases worldwide^1,2^. Poorly managed T2DM leads to various complications, including diabetic cardiomyopathy (DCM), a major cause of end-stage heart failure (HF), resulting in mortality and morbidity in patients with T2DM^3,4^. DCM is characterized by abnormal cardiac structure and function in diabetic patients without identified causes, such as coronary artery disease, uncontrolled hypertension, and significant valvular and congenital heart disease^5,6^. A wide variety of mechanisms have been suggested to be involved in this clinical condition in recent years, including cardiomyocyte oxidative stress, inflammation, fibrosis, various forms of cell death, mitochondrial dysfunction, and alterations in myocardial energetics^7–10^. However, the underlying mechanism has not yet been elucidated, and there is no specific therapy for DCM patients in clinical practice.

The main energy source of the adult heart is obtained from fatty acid (FA) oxidation, which accounts for 60–80%, with the remaining part being derived from glucose, lactate, and ketones^11^. However, in obese and diabetic patients, excessive accumulation of lipids and overactivation of lipid signaling pathways disturb the balance of FA uptake, metabolism, and oxidation in cardiomyocytes, leading to an increased flux of nonoxidative metabolic FAs and the accumulation of cardiotoxic fatty acid metabolites, thereby resulting in lipotoxic cardiomyopathy^12,13^. This condition not only changes cardiac metabolism but also causes the accumulation of damaged mitochondria and cardiac fibrosis^14–16^. Inhibiting FA utilization is a promising strategy for increasing cardiac efficiency in DCM.

Dipeptidyl peptidase-4 (DPP-4) inhibitors are an approved treatment for T2DM. Several studies have shown that they have a beneficial effect on cardiovascular diseases associated with metabolic syndrome and enhance blood pressure and vascular endothelial function^17–19^. Evogliptin® (EVO), a novel DPP-4 inhibitor, was developed by Dong-A, Republic of Korea, and approved as an oral antihyperglycemic drug for the treatment of T2DM by the Ministry of Food and Drug Safety of Korea in 2015. EVO has an effect on gluconeogenesis, hepatic steatosis, vascular inflammation, and whole-body composition^20–23^. However, the effect of DPP-4 inhibitors on HF is still unknown, and studies on EVO to understand its impact on diabetes and its complications, including DCM, are lacking. To explore these issues, we investigated the effects of EVO on DCM in db/db mice.

## Materials and methods

### Animal experiments

Eight-week-old male diabetic db/db mice (BKS.Cg-Dock7^m^+/+ Lepr^db^/J) and eight-week-old male nondiabetic db/m+ mice (C57BLKS/J) were purchased from Charles River (Japan). C57BKS/J mice were used as normal controls or wild-type (WT) mice. All animals were housed in a specific pathogen-free facility with controlled temperature (20–24 °C) and humidity (40–70%) on a 12 h light cycle with access to standard laboratory chow and tap water *ad libitum*. All experimental procedures were approved by the Inje Medical University Animal Care and Use Committee (approval No. 2011-049). After adaptive feeding for three days, WT and db/db mice with similar blood glucose levels and body weights were randomized into the following groups: untreated db/db control group and db/db+Evo group. Mice in the db/db+Evo group were intragastrically administered 100 mg/kg/day evogliptin (Dong-A, Korea) for 12 weeks, while WT and db/db control mice were administered equal amounts of 0.5% hydroxyethylcellulose (vehicle) via gavage. Body weight (BW) and food intake were measured weekly.

At the end of the treatment, the mice were sacrificed after performing LV catheterization under anesthesia with isoflurane (1.5% in 100% oxygen). Overnight-fasted mice were sacrificed by exsanguination under anesthesia by inhalation of 2% isoflurane in the ambient air of the room. Blood samples and heart tissue were collected for further experiments; the heart weight was also measured (Supplementary Fig. 1).

### Echocardiography

The mice were kept warm on a heating pad while under anesthesia (1–2% isoflurane supplemented with 100% oxygen), and transthoracic echocardiography was performed. Images were acquired using a high-frequency ultrasound system (Vivid 7, GE Healthcare, Korea) to detect cardiac structure alterations and cardiac function in vivo using an echocardiogram with an i13L-14 MHz probe. Additionally, 2-D guided M-mode images of the LV in parasternal short-axis views at the papillary muscle level were recorded. The left ventricular internal diameter in diastole/systole (LVIDd/s), end-diastolic volume (EDV), end-systolic volume (ESV), stroke volume (SV), ejection fraction (EF), and fractional shortening (FS) were obtained in conscious experimental animals. According to Teichholz, EDV = (7.0/(2.4 + LVIDd)) × LVIDd^3^) and ESV = (7.0/(2.4 + LVIDs)) × LVIDs^3^) were used to calculate the EF as follows: EF = 100 × (EDV – ESV)/ESV. LV diastolic function was obtained using the transmitral inflow velocities recorded with pulsed-wave Doppler in the apical 4-chamber view, and the longitudinal component of myocardial contraction was assessed with tissue Doppler imaging with the sample volume placed at the base of the posterior wall in the parasternal long axis. The ratio of the peak velocity of early to late filling of mitral inflow (E/A) and of early diastolic myocardial relaxation (e’) to active atrial contraction in late diastole (a’), (e’/a’), E/e’, and deceleration time (DT) were calculated. All data and images were saved and analyzed using an EchoPAC PC (GE Healthcare). Three or more consecutive cardiac cycles were averaged for all analyses.

### Fasting blood glucose and glucose tolerance test

Fasting blood glucose levels were measured in all mice after fasting for 16 h, once every two weeks during the treatment. Briefly, 2 μL of blood from the tail vein was collected via tail clipping and measured directly using a glucose meter (ACCU-CHEK; Roche, USA) with test strips. For the intraperitoneal glucose tolerance test (IPGTT), mice were fasted for 16 h prior to intraperitoneal injection of a 20% D-glucose solution. Blood glucose levels were determined before the injection (time 0) and at different time points after injection (30, 60, 90, 120, and 150 min).

### Blood sample collection and biochemical analyses

At the end of the study, the mice were anesthetized by inhalation of 5% isoflurane. Fasting blood specimens were collected from the heart in commercial tubes containing K3EDTA as an anticoagulant, and tubes without anticoagulants were used to collect the serum by centrifugation for 5 min at 1000 × *g*. The plasma and serum were stored at −20 °C until analysis. HbA1c, insulin, fructosamine, *creatine kinase-MB* (CK-MB), total cholesterol (TC), triglycerides (TGs), low-density lipoprotein cholesterol (LDL-C), high-density lipoprotein cholesterol (HDL-C), total lipid, and myoglobin levels were determined.

### Determination of TG levels in cardiac tissue

Heart samples were washed in phosphate-buffered saline (PBS) and homogenized in 1 mL of 5% Nonidet P 40 substitute solution (74385, Sigma‒Aldrich, Korea). The samples were heated at 80 °C for 3 min and then cooled to ~25 °C. The samples were then centrifuged for 3 min at 5000 × *g*. TG levels were determined using a triglyceride quantification kit (MAK266, Sigma-Aldrich, Korea) according to the manufacturer’s instructions.

### Histological analyses

The mouse hearts were excised, washed with PBS, and fixed in 10% paraformaldehyde. The hearts were then sent to Histoire (Ansan, Korea) for staining. To measure the cross-sectional and total area of LV cardiomyocytes, sections were stained with hematoxylin and eosin (HE) and photographed using a NanoZoomer Digital Slide Scanner (Hamamatsu, Japan). Each section was quantitatively measured using ImageJ 1.48 software (NIH, MD). For assessment of myocardial fibrosis, sections were stained by Masson’s trichrome method and photographed using NanoZoomer Digital Slide Scanners (Hamamatsu Photonics, Hamamatsu, Japan). The relative fibrotic area (% of the total area) was measured quantitatively using ImageJ 1.48 software.

### Electron microscopy analysis of in situ cardiac mitochondria

The dissected heart fragments (1 mm^3^) from experimental animals were fixed in 2.5% glutaraldehyde in PBS solution at 4 °C overnight and then with 1% osmium tetroxide in PBS for 2 h. The tissues were washed, dehydrated, and embedded, from which semithin sections were cut (0.5–1 µm). Ultrasectioning (60–90 nm) was performed, and the slices were double-stained with uranyl acetate and lead citrate and imaged using a JEM 1200 EX2 electron microscope (Jeol, Japan). The developed images were scanned on a flatbed scanner (Umax PowerLook 1100; Fremont, CA, USA) and analyzed using ImageJ software.

### Permeabilized left ventricle tissue oxygen consumption

Oxygen consumption was determined polarographically using a fiber optic oxygen monitor (Instech, Plymouth Meeting, PA, USA). The cardiac fibers were separated in a small Petri dish filled with buffer (7.23 mM K_2_EGTA, 2.77 mM CaK_2_EGTA, 20 mM imidazole, 0.5 mM DTT, 20 mM taurine, 5.7 mM ATP, 14.3 mM PCr, 6.56 mM MES, with 100 µL of 0.5 M EGTA; pH 7.1) on top of a frozen block under the dissecting scope. Next, ~2–3 mg (wet weight) of the fiber bundles was weighed. They were placed in saponin tubes for 30 min on a rotating tray in the refrigerator and then in washing buffer (105 mM K-MES, 30 mM KCl, 10 mM KH_2_PO4, 5 mM MgCl_2_, 0.5 mg/mL BSA, pH 7.1) for 15 min. Next, 2.1 mL of assay buffer was added to each chamber. Oxygen consumption was measured in an air-saturated (220 nmol O_2_/mL) respiration medium. The state 4 respiratory rate was determined in the presence of 5 mM malate, 5 mM glutamate, and 5 mM succinate with rotenone as the respiratory substrate for complex I and complex II. The state 3 (active) respiration rate was determined in the presence of 0.1 mM ADP. Oxygen uptake was expressed as ng-at O/min mg protein. Data were acquired at a sample rate of 100 ms and analyzed using the OOIsensors program (Ocean Optic Inc., FL, USA). The respiratory control ratio (RCR) was calculated as the ratio of the state 3/state 4 oxygen consumption rates.

### Western blotting analysis

Total protein was extracted from WT and db/db cardiac tissues using RIPA lysis buffer containing a protease and phosphatase inhibitor cocktail (Thermo Fisher, Waltham, MA, USA). The extracted proteins were separated by sodium dodecyl sulfate-polyacrylamide gel electrophoresis and transferred to polyvinylidene fluoride membranes. After blocking with 5% skim milk in TBS with 0.1% Tween 20, membranes were incubated with the following primary antibodies overnight at 4 °C: primary antibodies were purchased from Cell Signaling (TGF-β1, PGC1a, NRF1, NRF2, total OXPHOS, ACC, p-ACC Ser^79^, ACSL1, ATGL, FOXO1, p-FOXO1 Ser^256^, AMPK, p-AMPK Thr^172^, Akt, p-Akt Ser^473^, mTOR, p-mTOR Ser^2448^, PPARγ1/2, NF-κB p65, β-Actin, and GAPDH); Abcam (GLUT4, Col1a1, and TFAM); Invitrogen (FABP3, IGFBP7, and p-SREBP1 Ser^338^); and Santa Cruz Biotechnology (CD36, SREBP1, MFN1, OPA1, and DRP1). After washing, the membranes were incubated with the HRP–conjugated secondary antibody (Jackson ImmunoResearch, USA), diluted in 5% skim milk, and incubated for 1 h at room temperature. Finally, the membranes were washed with Tris-buffered saline (TBS) containing 0.1% Tween 20. Immunodetection was performed using an enhanced luminol-based chemiluminescent substrate (WESTSAVE Up, AbFrontier, Korea). GAPDH and β-Actin were used as loading controls. Quantification of each band was performed using ImageJ software.

### Quantitative real-time PCR

Total RNA from WT and db/db mouse cardiac tissues was extracted using TRIzol (Invitrogen, Carlsbad, CA, USA). Then, 1.5 µg of RNA was reverse-transcribed to cDNA using a Revert Aid First Strand cDNA Synthesis kit (Thermo Fisher) following the manufacturer’s protocol. The primers were obtained from Cosmo Genetech (Korea), and their sequences are listed in Supplementary Table 1. SYBR premix Ex Taq (Takara, Shiga, Japan) was used to perform real-time PCR with reactions prepared according to the manufacturer’s protocol (Bio-Rad, Hercules, CA, USA). All reactions were performed in triplicate. cDNA was amplified by 45 cycles using the following settings: 15 s at 95 °C, 30 s at 58 °C, and 30 s at 72 °C. The analysis was carried out using CFX Manager™ software (Bio-Rad, Hercules, CA, USA) and Microsoft Excel. The relative RNA levels were normalized to those of GAPDH. Primer sequences used for PCR are listed in Supplementary Table 1.

### Statistical analysis

Statistical analyses were performed using GraphPad Prism 8.0.1 software (San Diego, CA, USA). All data are expressed as the mean ± standard deviation (SD) or standard error (SE). Unpaired Student’s *t* test was used for comparisons between two groups, and one- or two-way analysis of variance (ANOVA) and post hoc Tukey’s test were used for multiple comparisons. Statistical significance was set at *p* < 0.05.

## Results

### Effects of EVO on body weight, blood glucose, HbA1c levels, food intake, and biochemical characteristics of diabetic mice

**Fig. 1:**
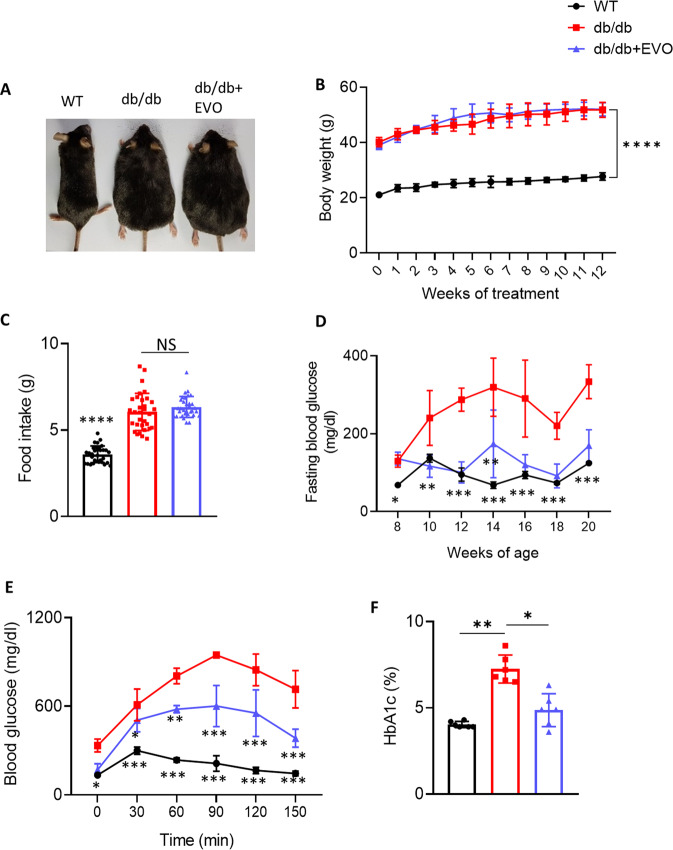
Effects of EVO on body weight, blood glucose and HbA1c levels, and food intake in db/db mice. **A** Morphology of WT, db/db, and db/db+EVO mice. **B** Weekly body weight (*n* = 10/group). **C** Food intake and **D** fasting blood glucose level (*n* = 10). **E** Glucose tolerance test after 12 weeks of EVO treatment (*n* = 10/group). **F** HbA1c levels (*n* = 6). Data are presented as the mean ± SE; *p < 0.05, **p < 0.01, ***p < 0.001, ****p < 0.0001, ns not significant.

Body weight was measured every week, food intake was measured three times per week, and fasting blood glucose levels were determined before and after every two weeks of treatment. After 12 weeks of treatment, IPGTT was performed on the mice before sacrifice. Blood samples were collected, and biochemical features were analyzed (Supplementary Fig. 1). Comparisons of various physical and biochemical parameters among the three groups of mice are shown in Fig. 1 and Supplementary Table 2. The results showed that there was no significant difference in body weight and food intake between db/db and db/db+EVO mice (Fig. 1B, C). Eight-week-old db/db mice exhibited higher blood glucose levels than WT mice of the same age (*p* < 0.05; Fig. 1D). EVO effectively decreased fasting blood glucose levels in db/db mice after two weeks of treatment (Fig. 1D) and improved glucose homeostasis (IPGTT results) after 12 weeks of treatment (Fig. 1E). EVO treatment also significantly reduced HbA1c levels in db/db mice (*p* < 0.05; Fig. 1F). In addition, fructosamine, CK-MB, cholesterol, TG, total lipid, and myoglobin levels were slightly decreased in db/db+EVO mice compared with db/db mice (*p* > 0.05; Supplemental Table 2). The results demonstrated that EVO had no effect on body weight and food intake in db/db mice but significantly decreased glucose and HbA1c levels.

### EVO treatment improved systolic and diastolic function in db/db mice

**Fig. 2:**
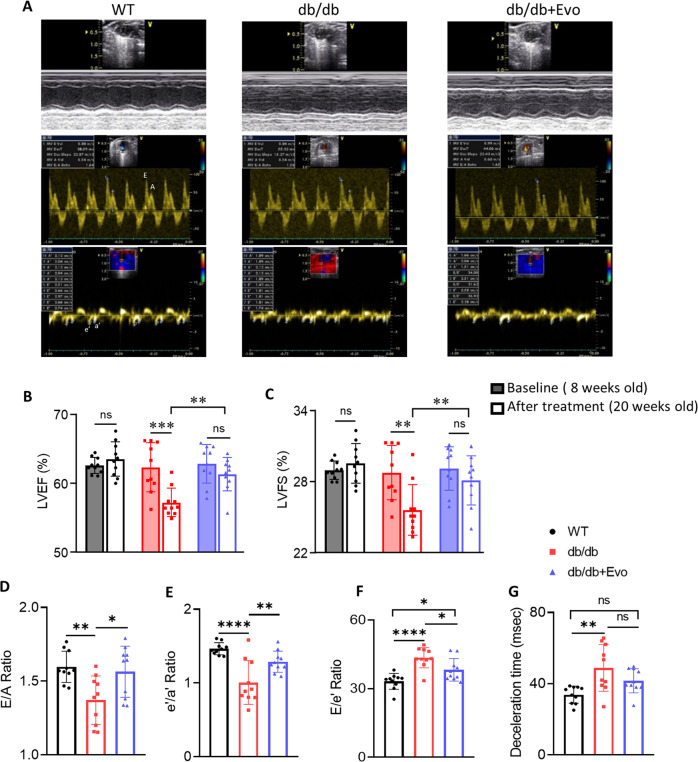
Effects of EVO on cardiac systolic and diastolic function in db/db mice. **A** Representative 2-D, M-Mode, and Doppler echocardiographic images of WT, db/db, and db/db+EVO mice. **B** Ejection fraction (EF%) and **C** fractional shortening (FS%). **D** Ratio of the velocities of early to late mitral flow (E/A). **E** Ratio of early diastolic myocardial relaxation to active atrial contraction in late diastole (e’/a’). **F** E/e’ ratio and **G** deceleration time (DT). Data are presented as the mean ± SE. *p < 0.05, **p < 0.01, ***p < 0.001, ****p < 0.0001, *n* = 10–20/group, ns not significant.

Echocardiographic data demonstrated that db/db mice exhibited a significant decrease in both systolic and diastolic function compared with that of WT mice (Fig. 2A), while db/db+EVO mice showed a remarkable improvement in EF and FS after 12 weeks of treatment (Fig. 2B, C). Doppler flow analysis revealed a decrease in the E/A and e’/a’ ratios as well as an increase in the E/e’ ratio, indicating that LV diastolic function was notably impaired in db/db mice, while EVO significantly reversed these diastolic function parameters after 12 weeks of treatment (*p* < 0.05, *p* < 0.01, and *p* < 0.05, respectively; Fig. 2D–F). In addition, DT was significantly increased in the db/db group compared to the WT group (*p* < 0.01; Fig. 2G), but EVO treatment resulted in an attenuation of DT compared to the WT group. Collectively, db/db mice developed marked LV systolic and diastolic dysfunction, but these were significantly reversed by EVO treatment.

### EVO treatment reduced cardiac hypertrophy and fibrosis in db/db mice

**Fig. 3:**
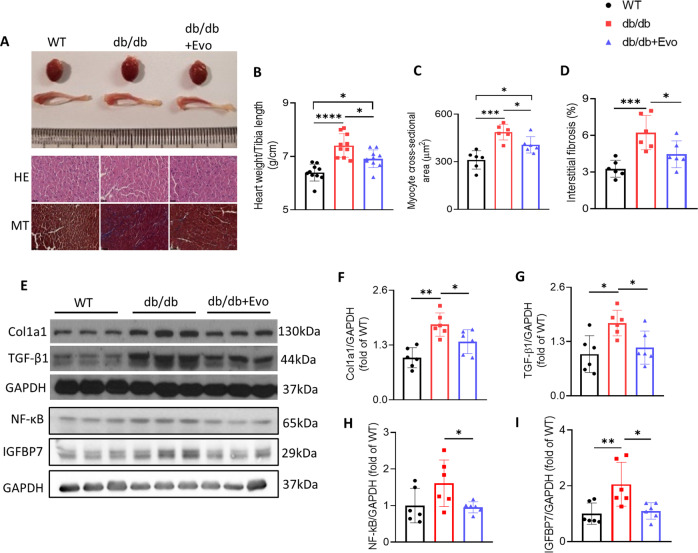
Effects of EVO treatment on cardiac hypertrophy and fibrosis in db/db mice. **A** Representative images of whole hearts and tibias from mice in different groups and hematoxylin and eosin- and Masson-stained heart sections. Magnification ×400. The scale bar = 100 µm. **B** The ratio of body weight/tibia length in different weeks of treatment (*n* = 10/group). **C** Myocyte cross-sectional area (µm^2^). **D** Interstitial fibrosis. **E**–**I** Protein expression and quantitative analysis of Cola1, TGF-β1, IGFBP7, and NF-κB in the hearts of mice as determined by western blotting. Data are presented as the mean ± SE. *p < 0.05, **p < 0.01, ***p < 0.001, ****p < 0.0001 (*n* = 6/group).

The hearts of db/db mice were clearly larger, and the heart weight to tibia length ratio was higher than those in WT mice (*p* < 0.0001; Fig. 3A, B). In addition, cardiomyocytes were clearly striated and regularly arrayed in WT mice, while myocardial fibers were disordered and myocyte cross-sectional area was increased in db/db mice, as shown by HE staining (*p* < 0.001; Fig. 3A, C). However, these features were also attenuated in the hearts of EVO-treated db/db mice (*p* < 0.05; Fig. 3B, C). Moreover, Masson’s staining showed that the interstitial fibrosis and accumulation of collagen fibers were increased in the hearts of db/db mice compared with those in WT mice (*p* < 0.001; Fig. 3A, D), and these parameters were significantly attenuated after 12 weeks of EVO treatment. To further verify these cardiac changes, we detected the protein expression of Col1a1 and TGF-β1 using western blotting analysis. The results showed that the expression of these proteins was increased in the hearts of db/db mice and was significantly attenuated by EVO treatment (*p* < 0.05; Fig. 3E–G). Additionally, insulin-like growth factor binding protein-7 (IGFBP7) has been defined as a biomarker of cardiac fibrosis^24^, and it has been reported that the expression of IGFBP7 is upregulated by TGF-β^25^. We found that IGFBP7 expression was elevated in db/db hearts and was significantly reduced in EVO-treated mouse hearts (*p* < 0.05; Fig. 3E, H). Moreover, NF-κB expression, which is downregulated by PGC1α and plays an important role in inflammation and cardiac hypertrophy, was attenuated by EVO treatment (*p* < 0.05; Fig. 3E, I). In summary, these results demonstrated that EVO treatment inhibited cardiac hypertrophy and fibrosis in db/db mice.

### EVO treatment attenuated myocardial mitochondrial injury and improved mitochondrial function in db/db mice

**Fig. 4:**
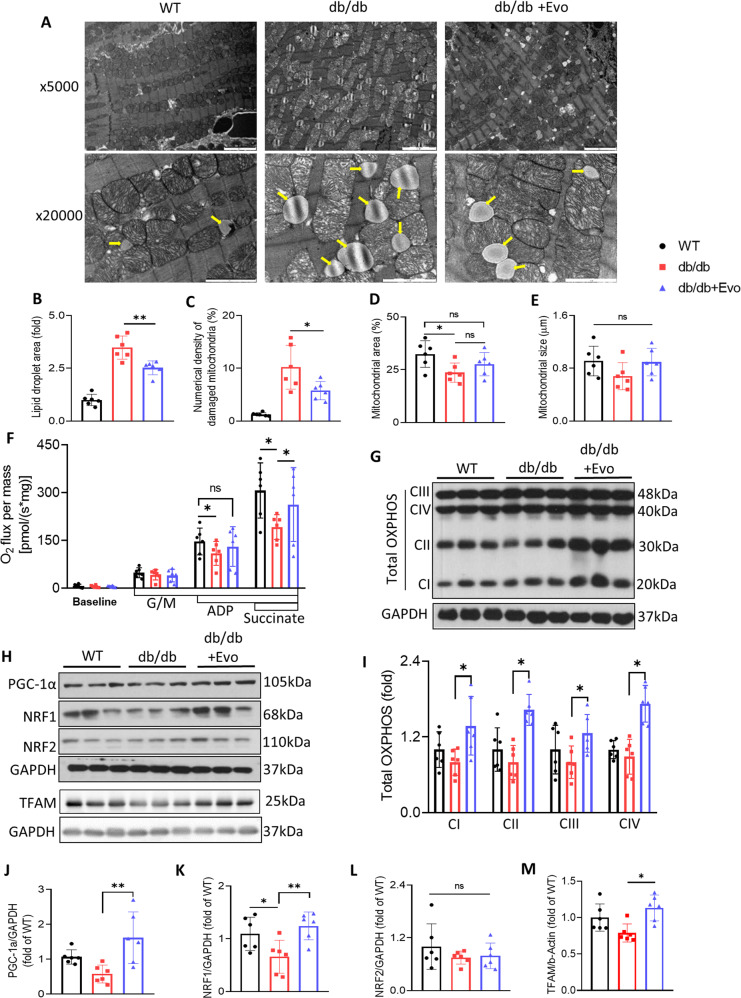
EVO treatment attenuated mitochondrial injury and the deposition of lipid droplets and modulated the PGC1α/NRF1/TFAM signaling pathway in the hearts of db/db mice. **A** Representative transmission electron microscopy images of left ventricular cardiac tissues, scale bar = 1 μm. **B**–**E** Lipid droplets, percentage of damaged mitochondria, and mitochondrial area and size (*n* = 4/group). **F** Saponin-permeabilized cardiac fiber oxygen consumption rate. **G**, **H** Protein expression of OXPHOS complexes I, II, III, PGC-1α, NRF1, NRF2, TFAM, and GAPDH in the hearts of mice after 12 weeks of treatment was determined by western blotting. **I**–**M** Quantitative analysis of these proteins. Data are presented as the mean ± SE. *p < 0.05, **p < 0.01, ns not significant (*n* = 6/group).

Transmission electron microscopy revealed clear evidence of mitochondrial injury. Representative images of the cardiac mitochondrial ultrastructure are shown in Fig. 4A. The results showed that EVO attenuated myocardial mitochondrial injury and the accumulation of lipid droplets, which contributed to decreased cardiac lipotoxicity in db/db mice (*p* < 0.01 and *p* < 0.05, respectively; Fig. 4B, C). The mitochondrial number and area were significantly decreased in diabetic cardiomyocytes compared with those in WT mice, but they were slightly increased in the db/db+EVO group (Fig. 4D and Supplementary Fig. 2c). There was no significant difference in mitochondrial size between the db/db and db/db+EVO groups (Fig. 4E). In addition, EVO increased the protein levels of complexes I, II, III, and IV in the electron transport chain of the mitochondria and enhanced the mitochondrial oxygen consumption rate in db/db mice (Fig. 4F, G, I). On the other hand, the PGC1α/NRFs/TFAM signaling pathway plays an important role in mitochondrial biogenesis^26^, and we next determined whether it was affected by EVO treatment. The present study showed that EVO treatment significantly prevented the impaired expression of PGC1α, NRF1, and TFAM in the hearts of db/db mice (*p* < 0.05; Fig. 4H, J–M). However, there was no significant difference in NRF2 expression between the db/db and db/db+EVO hearts (*p* > 0.05; Fig. 4L), and AMPK phosphorylation, the major upregulated factor upon PGC1α posttranslation modification, was not changed in EVO-treated hearts compared with db/db hearts (*p* > 0.05; Supplementary Fig. 2a, b). Additionally, we assessed mitochondrial dynamics, which is controlled mainly by the two opposing processes of fission and fusion. The results showed that the markers for fusion, namely, mitofusin 1 (MFN1) and optic atrophy protein 1 (OPA1), were slightly augmented in hearts from db/db mice treated with EVO compared with the db/db control group. In contrast, the marker for fission, that is, dynamin-related protein 1 (DRP1), was slightly reduced. However, there were no significant differences in these characteristics between the db/db and db/db+EVO hearts (*p* > 0.05; Supplementary Fig. 2d, e–g). In summary, EVO can prevent mitochondrial damage and enhance mitochondrial function via the PGC1α/NRF1/TFAM signaling pathway in the hearts of db/db mice.

### EVO treatment alleviates cardiac lipotoxicity in db/db mice

**Fig. 5:**
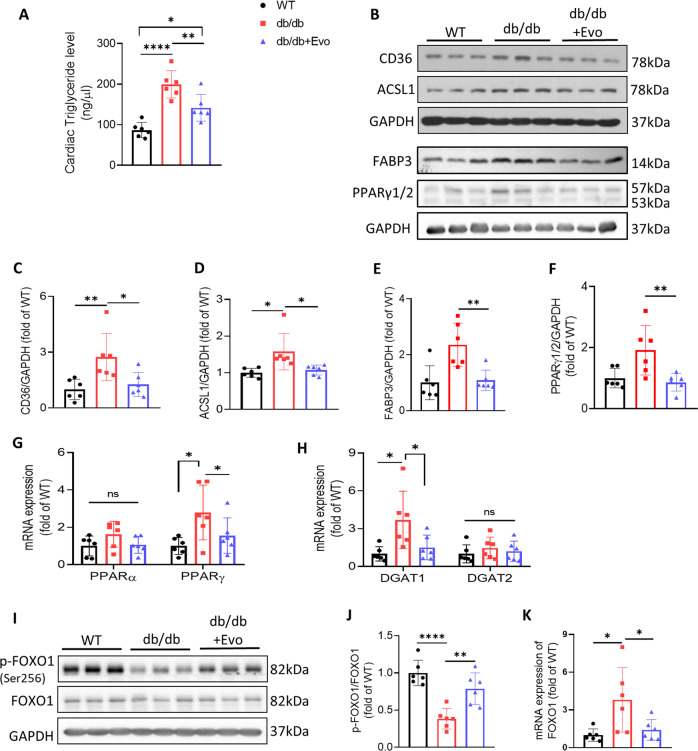
EVO treatment alleviates cardiac lipotoxicity in db/db mice. **A** Cardiac triglyceride level. **B** Protein expression of CD36, ACSL1, FABP3, PPARγ1/2, and GAPDH using western blotting analysis. **C**–**F** Quantitative analysis of these proteins. **G**, **H** mRNA expression of PPARα, PPARγ, DGAT1, and DGAT2 in the hearts was determined by RT‒PCR. **I** Protein expression of FOXO1 (total and phosphorylated). **J**, **K** Quantitative analysis of FOXO1 by western blotting and RT‒PCR. Data are presented as the mean ± SE. *p < 0.05, **p < 0.01, ****p < 0.0001, ns not significant (*n* = 6/group).

Next, the lipotoxic status of the hearts was evaluated. Cardiac TG levels were significantly increased in the myocardium of db/db mice compared to WT and db/db+EVO mice (*p* < 0.0001 and *p* < 0.01, respectively; Fig. 5A). EVO was also shown to effectively reduce the accumulation of lipid droplets in db/db hearts, as shown in Fig. 4A and Fig. 4B. This means that EVO reduces lipid accumulation in T2DM hearts, leading to the amelioration of cardiac lipotoxicity. A wide variety of metabolic pathways and target proteins are involved in the regulation of cardiomyocyte lipid levels and their signaling, which have, in turn, been implicated in the development of lipotoxic cardiomyopathy^12^. The western blotting results showed that CD36, a major cardiac FA transporter, was significantly enhanced in the hearts of T2DM db/db mice compared with those of WT and db/db+EVO mice (*p* < 0.01 and *p* < 0.05, respectively; Fig. 5B, C). Overexpression of CD36 in the cardiomyocytes of diabetic db/db mice promotes CD36-mediated long-chain FA uptake and TG accumulation, inducing diabetes-mediated cardiac lipotoxicity and subsequent contractile dysfunction^12^. Additionally, EVO treatment significantly reduced ACSL1 expression (Fig. 5B, D). Moreover, we found that the expression of fatty acid binding protein 3 (FABP3), a chaperone for lipids and a biomarker for heart injury, was obviously increased in the myocardium of db/db mice compared with the db/m group, while EVO significantly reduced its expression (*p* < 0.01; Fig. 5B, E). Our study further showed that EVO treatment decreased PPARγ expression in db/db hearts but did not affect PPARα gene expression (Fig. 5B, F, G). In addition, the results of real-time PCR indicated a statistically significant increase in the mRNA expression of diacylglycerol O-acyltransferase 1 (DGAT1) in db/db mice (*p* < 0.05; Fig. 5H), but there was no difference in DGAT2 expression among the groups (*p* > 0.05, Fig. 5H). However, ACC phosphorylation was significantly decreased in diabetic cardiomyocytes compared with that in WT cardiomyocytes (*p* < 0.05; Supplementary Fig. 3a, b), while EVO slightly increased ACC phosphorylation. No significant differences in ATGL expression were observed among the groups (*p* > 0.05; Supplementary Fig. 3a, c). Because of the shift in cardiac substrate utilization from glucose to FAs and concomitant FA overload, which is also a consequence of cardiac lipotoxicity, we examined GLUT4 expression and found that EVO tended to increase the expression of GLUT4 (Supplementary Fig. 3a, d, e).

Because forkhead box protein O 1 (FOXO1) activation recruits the FA translocase CD36 to the plasma membrane and increases FA uptake and oxidation^27^, we further explored whether EVO markedly decreased the lipid content via FOXO1 activity. The results showed that EVO reduced FOXO1 gene expression and inhibited FOXO1 activation by increasing its phosphorylation (*p* < 0.05 and *p* < 0.01, respectively; Fig. 5I–K).

On the other hand, we found that EVO did not regulate insulin-mediated signaling through the SREBP-1c gene, which is involved in lipogenesis in the liver and kidney. Although SREBP-1c expression was significantly increased in db/db mice compared with db/m mice (Supplementary Fig. 4a, b, e), there was no significant difference in this signal between db/db mice and EVO-treated db/db mice (Supplementary Fig. 4c, d–f). However, it tended to be attenuated in EVO-treated db/db hearts (Supplementary Fig. 4d).

Collectively, we suggest that changes in lipid metabolism in the hearts of db/db EVO-treated mice are cardiac-specific effects of EVO rather than systemic effects.

**Fig. 6:**
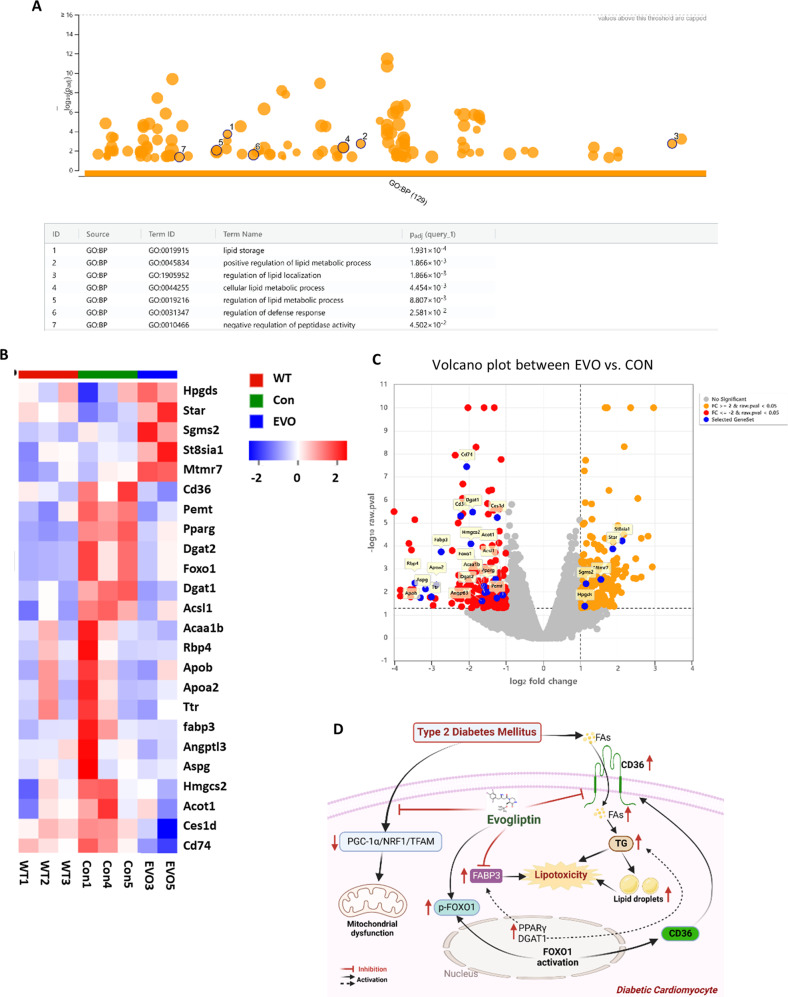
RNA sequencing and systemic analysis of EVO target genes. **A** Based on GO analysis, EVO significantly changed biological processes related to lipid metabolism in the hearts of db/db mice. **B** Heatmap analysis of the change patterns of 24 genes related to cellular lipid metabolism in the WT, Con, and EVO groups. **C** Volcano plots of the EVO vs. Con group and changes in the gene expression patterns of 24 genes related to cellular lipid metabolism. **D** Graphical abstract – EVO ameliorated cardiac lipotoxicity via the inhibition of overactivated lipid signaling pathways in diabetic cardiomyocytes and improved mitochondrial function via the PGC1α/NRF1/TFAM signaling pathway.

To confirm this statement and further explore whether the improvement in lipotoxicity following EVO treatment was mainly due to the decrease in lipid influx, inhibition of cardiac lipid synthesis, or increase in efflux, RNA sequencing was conducted. A multidimensional scaling (MDS) analysis showed that the gene expression changes among the WT, db/db, and EVO treatment groups were clearly distinguished (Supplementary Fig. 5a). In addition, these groups expressed quantitative differences in gene expression associated with cardiomyopathy (Supplementary Fig. 5b). Importantly, through GO analysis, we determined that EVO significantly changed biological processes related to lipid metabolism in the hearts of db/db mice (Fig. 6A and Supplementary Fig. 5c). Furthermore, 24 genes associated with cellular lipid metabolism were enriched in the groups. It was confirmed that EVO changed the expression patterns of these genes based on the heatmap and volcano plots (Fig. 6B, C). In the EVO target protein map created through STRING protein network analysis, major genes related to lipid metabolism were located in the center of the entire network. This finding supports our suggestion that the major mechanism of the cardioprotective effect of EVO is through the regulation of lipid metabolism (Supplementary Fig. 5d).

In summary, these results indicate that EVO prevented the increase in cardiac TG levels and FA transport and inhibited the overactivation of lipid signaling pathways in diabetic cardiomyocytes, thereby attenuating cardiac lipotoxicity in db/db mice (Supplementary Fig. 5d).

## Discussion

In this study, we showed the cardioprotective effects of the DPP4 inhibitor EVO in diabetic db/db mice. EVO attenuated both systolic and diastolic dysfunction, cardiac hypertrophy, and fibrosis, which are the main etiologies of DCM. In addition, through additional experiments, we confirmed that EVO reduced mitochondrial dysfunction and lipotoxicity by reducing fat accumulation in the myocardium or upregulating transcription factors such as NRF1 and PGC1.

Heart failure and related morbidity and mortality are major challenges in the treatment of T2DM. Although hyperglycemia, insulin resistance, and impaired cardiac insulin metabolic signaling are associated with DCM, the pathogenesis of DCM remains complex, and there is no specific treatment to date^10,28^. Since the 2008 guidance document by the US Food and Drug Administration (FDA), all new glucose-lowering agents must demonstrate cardiovascular (CV) safety^29^. Therefore, a wide range of clinical trials have addressed the relationship between strict glycemic control and CV endpoints. Among them, sodium-glucose cotransporter type 2 (SGLT-2) and DPP-4 inhibitors are promising drugs for reducing the morbidity of CV diseases related to diabetes, as they have positive data published related to CV outcomes. In particular, DPP-4 inhibitors are not associated with hypoglycemia or weight gain and have a good safety profile; therefore, their role as a substitute for sulfonylurea is becoming important^30^. In addition, they can be prescribed to patients with moderate to severe chronic kidney disease.

Beyond the glucose-lowering effect, DPP-4 inhibitors positively affect vascular endpoints and other CV events^31,32^. Previous studies have explained the possible mechanism of the beneficial effects on body weight, blood pressure (without an increase in heart rate), postprandial lipemia, inflammatory markers, oxidative stress, and endothelial function in patients with T2DM^17,33^. However, cellular-level studies on the myocardium or mitochondria are lacking.

DPP-4 is widely distributed throughout most tissues of the body and acts as a plasma membrane enzyme^34^. It regulates postprandial glucose by rapidly inactivating GLP-1 and GIP^35^. Therefore, DPP4 inhibitors stimulate insulin secretion in a glucose-dependent manner and reduce glucagon secretion from the pancreas by enhancing the endogenous bioactive hormones, incretin, GLP-1, and GIP, which are responsible for the antihyperglycemic effect^35,36^.

Evogliptin, a new DPP-4 inhibitor developed by Dong-A company, was first approved as an oral antihyperglycemic drug for the treatment of T2DM. Several studies have determined the influence of EVO on liver gluconeogenesis, hepatic steatosis, vascular inflammation, and whole-body composition^21–23,37^. In addition, EVO directly induces fat loss through an increase in Ppargc1α in white adipose tissue in obese mice^23^, inhibits vascular inflammation via modulation of Sirt1/NF-κB in atherosclerosis in ApoE−/− mice^21^, and ameliorates fatty liver by increasing insulin sensitivity^22^. However, limited information is available regarding the direct effects of DPP4 inhibition with EVO on DCM. To explore these issues, the effects of EVO-induced DPP4 inhibition on the DCM model in db/db mice were investigated. We found that EVO treatment reduced blood glucose and HbA1c levels and improved glucose tolerance in db/db mice, consistent with the results of other studies^37–39^. However, in our study, EVO did not influence body weight gain, which differs from previous publications that reported that EVO decreased^20^ or increased body weight^40^. This difference may be due to the distinct research subjects, the age of the mice, or the dose of EVO. EVO treatment did not produce any significant changes in serum cholesterol, TG, HDL, LDL, and total lipid levels, which are indicators of systemic lipid metabolism in db/db mice. This finding indicates that EVO particularly impacts cardiac lipid metabolism and that these effects are not systemic.

In addition, although the early stage of DCM exhibited diastolic dysfunction, we found that EVO treatment improved both systolic and diastolic function by analyzing echocardiography data in db/db mice, including a significant increase in EF and FS, as well as the E/A and e’/a’ ratios, and a reduction in the E/e’ ratio compared with those of nontreated db/db mice.

Cardiomyopathies are characterized by cardiac hypertrophy, which can contribute to ventricular dysfunction in HF^41^. Myocardial fibrosis occurs as a result of the excessive accumulation of extracellular matrix proteins in the heart and type 1 collagen, mediated by TGFβ1, a well-known profibrotic factor^42^. This accumulation can worsen ventricular stiffness, leading to cardiac dysfunction. In the present study, EVO treatment reduced the heart weight/tibia length, myocyte cross-sectional area, interstitial fibrosis, and molecular markers of fibrosis, such as collagen 1 and TGFβ1 (Fig. 3). Recently, studies of chronic HF have shown that higher levels of IGFBP7 are associated with diabetes mellitus and obesity^43^. IGFBP7 has been defined as a biomarker of myocardial fibrosis and was shown to be upregulated by TGF-β^25^. In the present study, cardiac IGFBP7 expression was significantly decreased after EVO treatment.

Mitochondria are considered the powerhouses of cells. To maintain continuous pump function, the heart requires large amounts of high-energy phosphates and accounts for approximately 8% of the total ATP consumption of the body. Recent studies have implicated mitochondrial injury as a key factor in the pathophysiology of DCM^28^. Improvement in mitochondrial function is a target for potential therapeutic strategies in many diseases. Furthermore, the transcription factors NRF1 and NRF2 are master regulators of antioxidant and stress responses^44,45^. The PGC1a/NRFs/TFAM signaling pathway plays an important role in mitochondrial function^26^. The current study demonstrated that EVO treatment attenuated myocardial mitochondrial injury and enhanced the oxygen consumption rate in db/db mice, as well as elevated the expression of complexes I, II, III, and IV in the electron transport chain of mitochondria. Although there was no significant difference in NRF2 expression and AMPK phosphorylation between the db/db and db/db+EVO groups, EVO treatment significantly increased PGC1α, NRF1, and TFAM expression in the hearts of db/db+EVO mice compared to their expression in those of db/db mice. These results indicate that EVO enhanced mitochondrial function in db/db mice via PGC1α/NRF1/TFAM. In contrast to our findings, AMPK phosphorylation was increased in db/db hearts and decreased in those of sitagliptin (another DPP-4 inhibitor)-treated db/db mice at 10 weeks old, as shown in the study by M. Lenski et al.^46^. This difference could be due to the age of the animals and the fact that the db/db mice belonged to distinct stages of DCM development. AMPK is defined as the main upstream activator of PGC1α and plays an important role in the regulation of fatty acid oxidation and mitochondrial biogenesis^47^. However, the mechanism by which EVO induces the activation of AMPK or other upstream activators of PGC1α remains unknown, and further studies are needed.

Additionally, the p65 subunit of NF-κB can bind to PGC1α and is thereby associated with cardiac hypertrophy, inflammation, and metabolic disturbances in cardiac cells^48,49^. This study also confirmed that EVO attenuated cardiac NF-κB expression in db/db mice.

Next, we evaluated the lipotoxicity status underlying the effects of EVO in the mouse heart. In DCM, decreased glucose and increased FA utilization lead to lipid accumulation, resulting in lipotoxicity, oxidative stress, and mitochondrial dysfunction. The accumulation of lipids in the cardiac muscle in obesity and diabetes is accompanied by accelerated myocardial FA oxidation rates. Studies have shown that diet-induced obese mice and diabetic rats and insulin-resistant ob/ob and db/db mice have increased myocardial FA oxidation rates in parallel with reduced cardiac efficiency^50,51^. Likewise, we observed that lipid accumulation was increased in the hearts of diabetic mice, as shown by the enhanced cardiac lipid droplets and TG levels (Figs. 4A, B and 5A). Three proteins, namely, CD36, FATP4, and FABP3, play critical roles in the transmembrane trafficking of FAs in cardiomyocytes^52^. Among these, CD36-dependent mechanisms are important in the development of insulin resistance and, subsequently, in the pathogenesis of DCM^53^. FABP3 serves as an intracellular lipid chaperone that is critical for maintaining the homeostatic function of cardiac muscle^54^. The present study showed that CD36 and FABP3 were significantly increased in the hearts of db/db mice compared to WT mice, while EVO treatment significantly alleviated this increase after 12 weeks of treatment. EVO treatment also significantly reduced ACSL1 expression, which is considered a building block in the synthesis of TG and other complex lipids. Moreover, previous studies have demonstrated that mice with cardiac-specific overexpression of PPARα or PPARγ, the transcription factors involved in regulating lipid metabolism, exhibited a phenotype similar to that of DCM^55,56^. Our results showed that PPARγ mRNA expression was increased in db/db mouse hearts but was significantly decreased in db/db mice treated with EVO. In contrast, DGAT1, a downstream target of PPARs that contributes to the final step in TG synthesis, was attenuated by EVO treatment. Furthermore, PPARs are ligand-activated transcription factors associated with FA uptake and oxidation; increased PPAR expression can activate several target genes, such as FABP3, CD36, and FATP4, thereby increasing these processes^57^. PPARs in turn are upregulated by PGC1α. Additionally, PPARγ may be a direct target gene of SREBP-1c, a transcription factor that controls lipogenesis. Increased SREBP-1c leads to augmented PPARγ, resulting in an increase in lipogenesis. However, in the present study, EVO slightly attenuated SREBP-1c gene expression in db/db hearts, but there was no effect in the db/db liver and kidney.

A recent study suggested that impaired insulin signaling activates FOXO1 and increases PPARα expression in cardiomyocytes, causing oxidative stress and DCM^58^. Our results showed that EVO reduced FOXO1 gene expression and inhibited FOXO1 activation by increasing its phosphorylation. However, it did not affect PPARα mRNA expression. Previous studies indicated that CD36-mediated fatty acid uptake upregulated the activity of FOXO1^27^.

The RNA sequencing results showed that EVO significantly altered the biological processes related to lipid metabolism in the hearts of db/db mice. Among 24 detected genes that were related to cellular lipid metabolism and enriched in groups were CD36, FABP3, DGAT1, PPARγ, and FOXO1, which are described above. These results further confirm that the improvement in lipotoxicity by EVO was mainly due to the decrease in cardiac lipid synthesis and accumulation in db/db mice.

Taken together, our findings suggest that DCM is related to lipotoxicity and that EVO ameliorates cardiac lipotoxicity via the inhibition of overactivated lipid signaling pathways in diabetic cardiomyocytes. In addition, EVO preserves mitochondrial function by increasing the activity of the PGC1α/NRF1/TFAM signaling pathway (Fig. 6D) and inhibits the development of cardiac hypertrophy and fibrosis by attenuating TGFβ/IGFBP7/Col1a1 expression. However, this potential mechanism requires further investigation.

This is the first study to demonstrate that DPP-4 inhibition by evogliptin prevents diabetic cardiomyopathy by reducing cardiac lipotoxicity, mitochondrial damage, and fibrosis in db/db mice. The present study suggests that inhibiting fatty acid utilization via DPP-4 inhibition with evogliptin is a promising strategy to elevate cardiac efficiency in DCM.

## Supplementary information

Supplemental Material
