# Supplementary material for: Evogliptin, a DPP-4 inhibitor, prevents diabetic cardiomyopathy by alleviating cardiac lipotoxicity in db/db mice
Source: Exp Mol Med. 2023 Apr 3;55(4):767–78. doi: 10.1038/s12276-023-00958-6 (PMC10167305; doi:10.1038/s12276-023-00958-6)
Supplement: Supplementary file 1 — Supplemental Material [file 12276_2023_958_MOESM1_ESM.pdf]

Supplementary method

Design of animal experiments

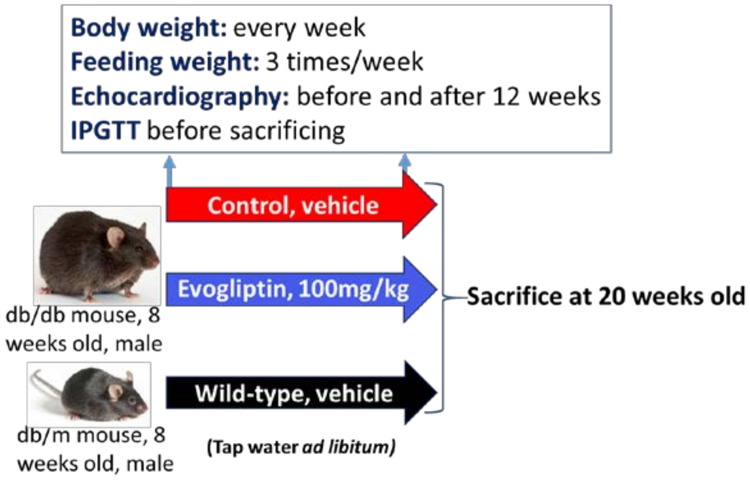

Supplementary Fig. 1. Design of animal experiments

RNA sequencing

Total RNA was extracted using the miRNeasy kit (Qiagen) under the protocols of the manufacturer. RNA-seq was performed for mouse samples of wild-type (WT), db/db control (Con), and db/db mice treated with Evogliptin (EVO). The quality was accessed by Bioanalyzer. Samples with high RNA integrity number (>8) were used for library construction. One hundred nanograms of total RNAs were used for each sequencing library. For the Illumina library preparations, the extracted RNAs was synthesized using TruSeq Stranded Total RNA Library Prep Gold Kit (Illumina Inc., CA, USA) and sequenced using Illumina HiSeq 2000. Paired-end reads were trimmed by quality score using Trimmomatic [1] and mapped to the mouse (*Mus musculus*) reference genome (mm10) using HISAT2 [2]. Gene expression levels were quantified by means of the reads per kilo base per million mapped reads (RPKM) method. Differentially expressed genes between different conditions were identified using the GFOLD algorithm [3]. Lowly expressed genes which were below 1 RPKM were discarded. Enriched biological pathways of differentially expressed genes (DEGs) in the con and Evo groups were analyzed using gProfiler (https://biit.cs.ut.ee/gprofiler/orth) [4]. System analysis such as MDS, heatmap, and volcano plot of DEGs was analyzed through Macrogen's DEG viewer program, and protein network analysis of DEGs was analyzed through STRING program [5].

Supplementary Table 1. Primers used for transcript quantification by RT-PCR

Gene		Primer sequences (5’-3’)
DGAT1	F	TCCGTCCAGGGTGGTAGT
	R	TGAACAAAGAATCTTGCAGACGA
DGAT2	F	GCGCTACTTCCGAGACTACTT
	R	GGGCCTTATGCCAGGAAACT
PPARalpha	F	TCGAGGAAGGCACTACACCT
	R	TCTTCCCAAAGCTCCTTCAA
PPARgamma	F	ACGATCTGCCTGAGGTCTGT
	R	CATCGAGGACATCCAAGACA
SREBP-1c	F	CACTCAGCAGCCACCATCTA
	R	GCTGTCAGCAGCAGTGAGTC
GAPDH	F	AACTTTGGCATTGTGGAAGG
	R	ACACATTGGGGGTAGGAACA

F, forward primer; R, reverse primer

Supplementary Table 2. Blood component analysis in EVO-treated db/db mice

	WT	db/db	db/db+EVO
Fructosamine (µmol /L)	262.5±20.0**	512.0±215.5	467.6±134.2
CK-MB (ng/mL)	0.22±0.05	0.27±0.04	0.25±0.03
Cholesterol (mg/dL)	75.8±11.5**	105.6±16.9	108.1±34.3
Triglyceride (mg/dL)	14.8±4.8*	40.3±19.1	36.7±10.7
HDL/C (mg/dL)	51.8±10.8	67.0±7.3	65.7±9.2
LDL/C (mg/dL)	5.85±1.07*	8.00±3.27	8.6±4.1
Total lipid (mg/dL)	296.2±64.5**	499.2±94.5	473.8±111.2
HbA1c (%)	3.98±0.2**	7.04±0.92	5.45±1.38#
Insulin (uU/mL)	0.40±0.1	0.33±0.18	0.32±0.12
Myoglobin (ng/mL)	16.1±5.7*	27.9±10.4	18.2±8.7

*P<0.05 vs. db/db and db/db+EVO

**P<0.01 vs. db/db and db/db+EVO

#P<0.05 vs. db/db

Data are means ± standard deviation (n=6/group)

Supplementary Fig. 2

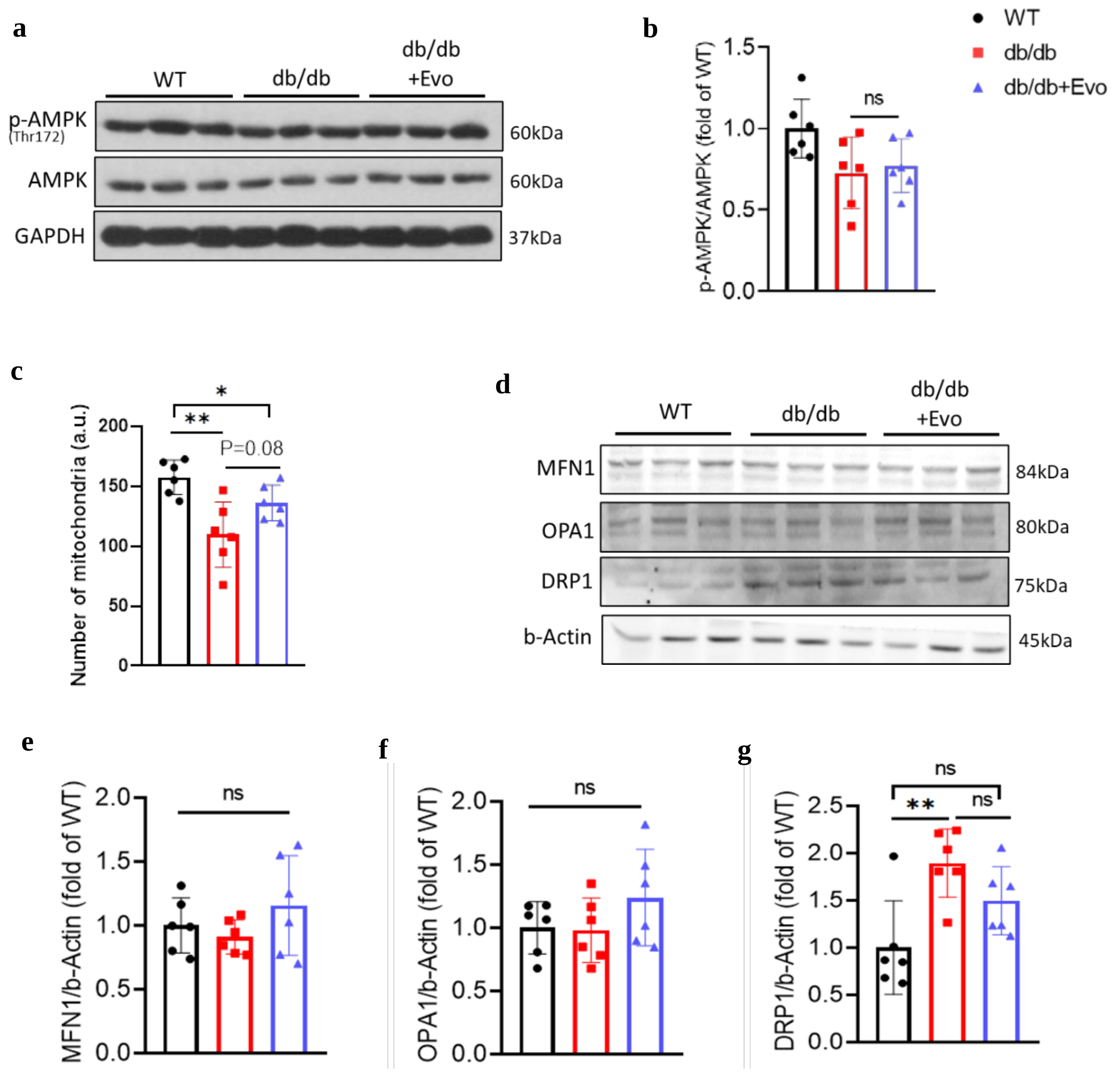

Supplementary Fig. 2: EVO treatment did not affect AMPK phosphorylation and Mitochondrial dynamics in db/db hearts. (a) Protein expression of AMPK (total and phosphorylation) and GAPDH, (b) Quantitative analysis of these proteins. (c) Number of mitochondria, (d) Protein expression of MFN1, OPA1, DRP1 and b-actin. (e-g) Quantitative analysis of these proteins. Data are presented as the mean±SE. *p<0.05, **p<0.01, ns: not significant (n=6/group).

Supplementary Fig. 3

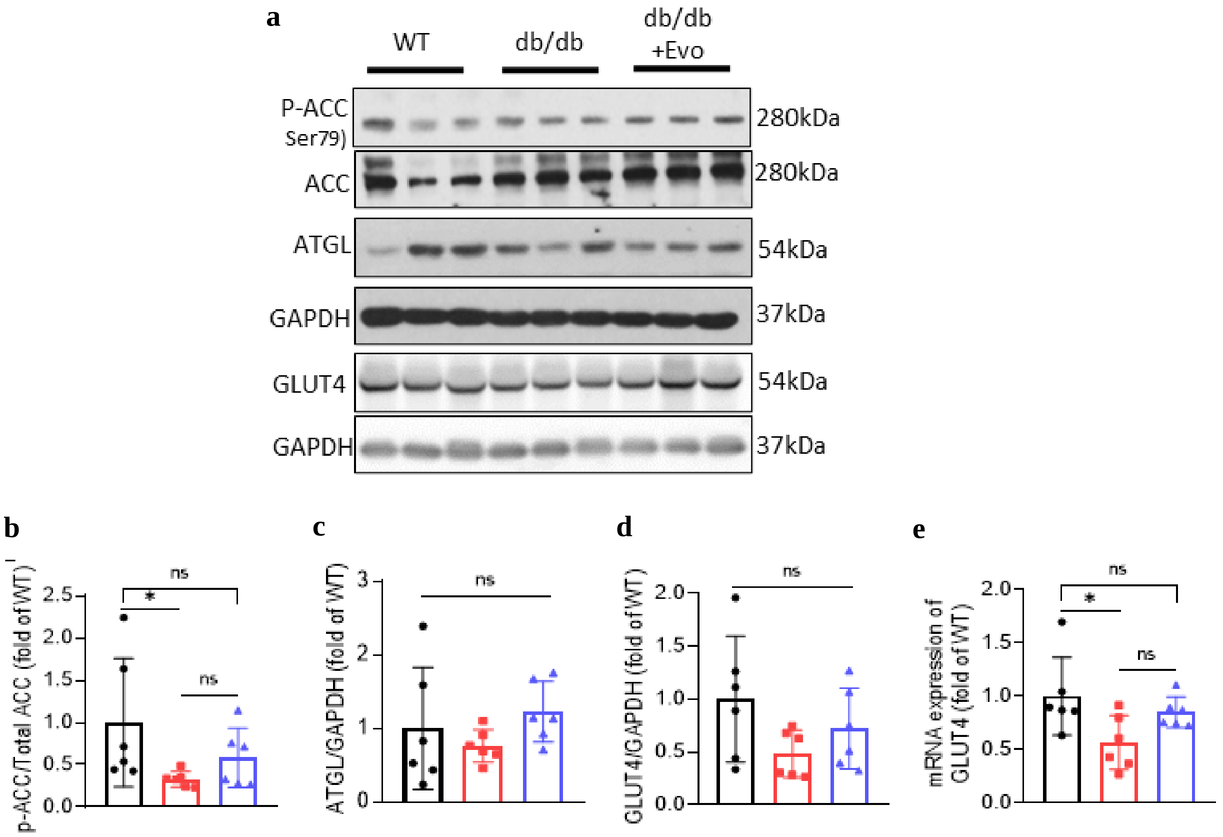

Supplementary Fig. 3: EVO treatment did not affect ACC phosphorylation and ATGL expression in db/db hearts, but it tended to increase the expression of GLUT4. (a) Protein expression of ACC (total and phosphorylation), ATGL, GLUT4 and GAPDH, (b-d) Quantitative analysis of these proteins, (e). mRNA expression of GLUT4 in the heart were determined by real-time PCR. Data are presented as the mean±SE. *p<0.05, ns: not significant (n=6/group).

Supplementary Fig. 4

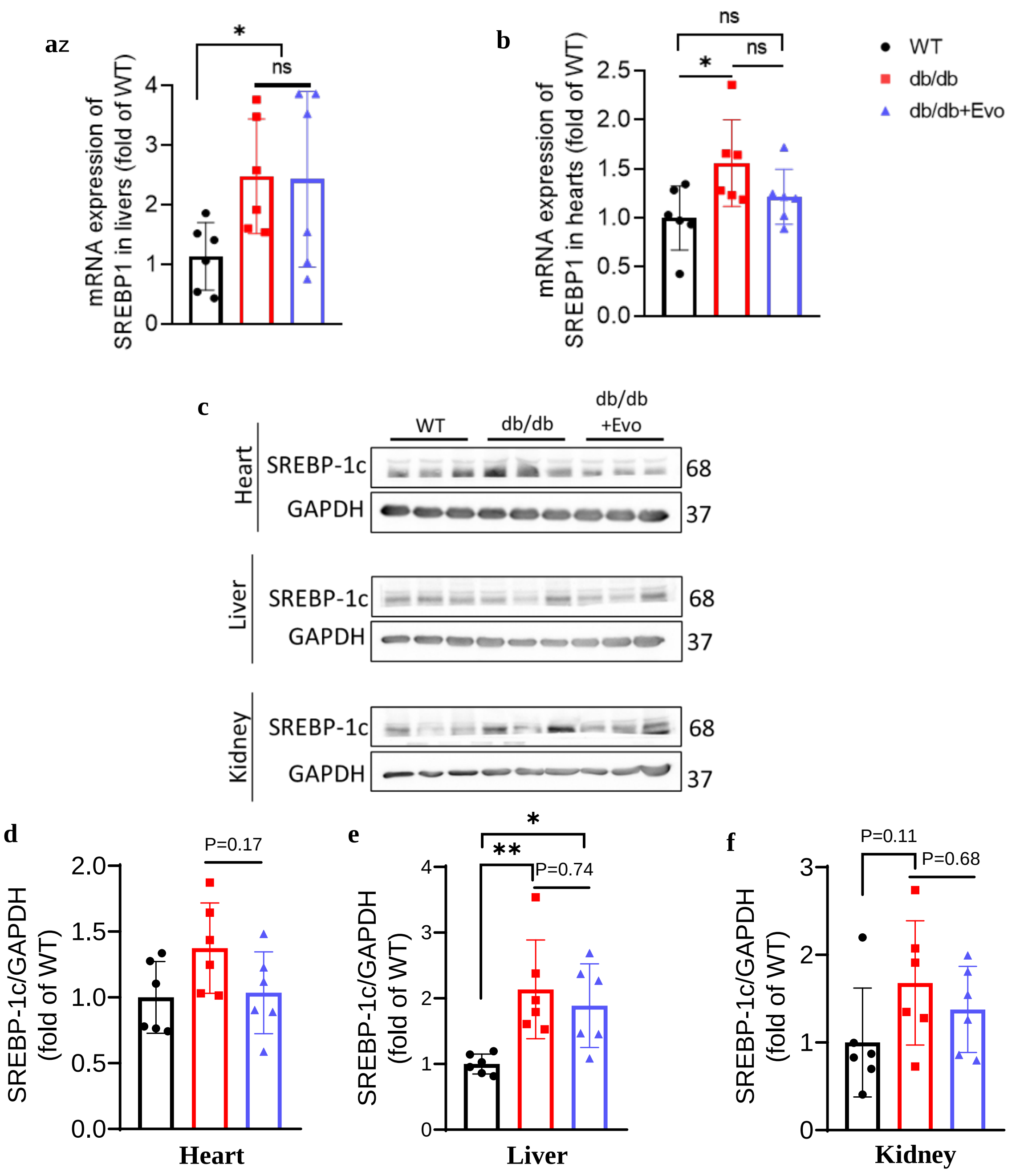

Supplementary Fig. 4: EVO treatment did not affect mRNA expression of SREBP-1c in heart, liver and kidney of db/db mice. (a) mRNA expression of cardiac SREBP-1c. (b) mRNA expression of SREBP-1c in liver. (c) Protein expression of SREBP-1c in heart, liver and kidney. And (d-f) Quantitative analysis of this protein in heart, liver and kidney, respectively. Data are presented as the mean±SE. **p*<0.05, ***p*<0.01, ns: not significant (n=6/group).

Supplementary Fig. 5

a

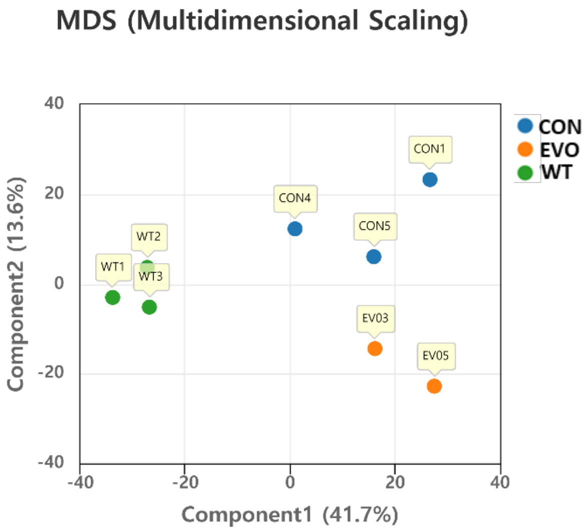

b

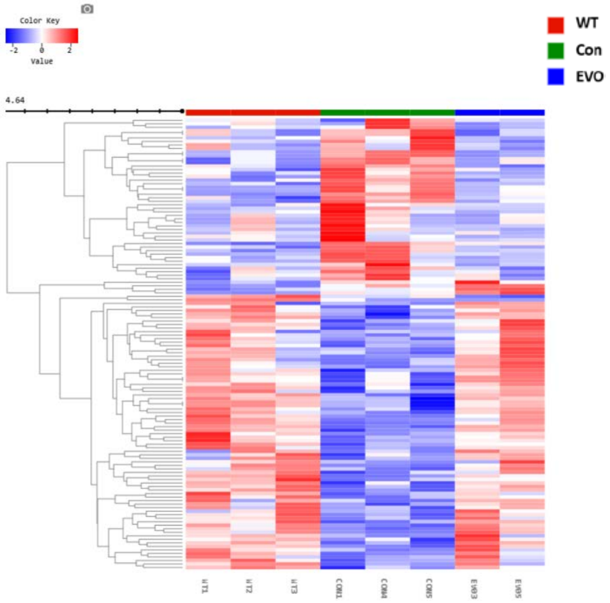

c

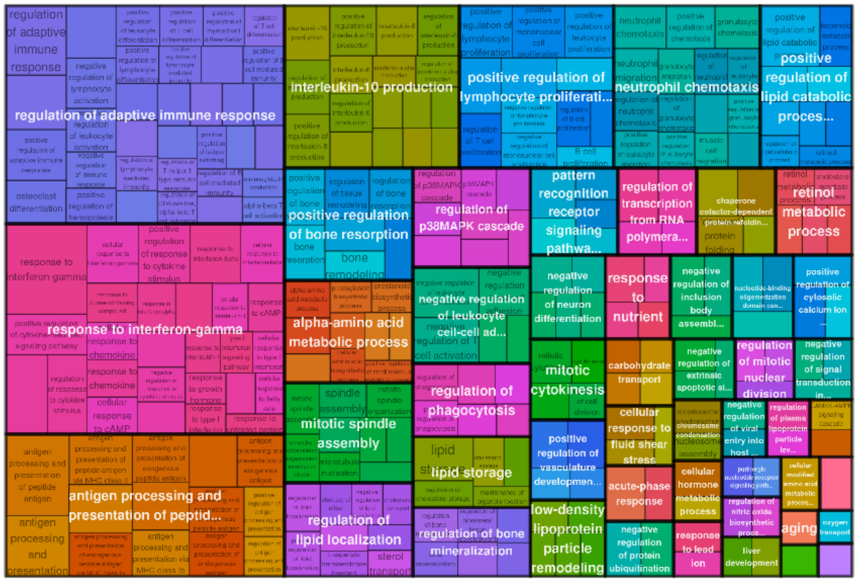

d

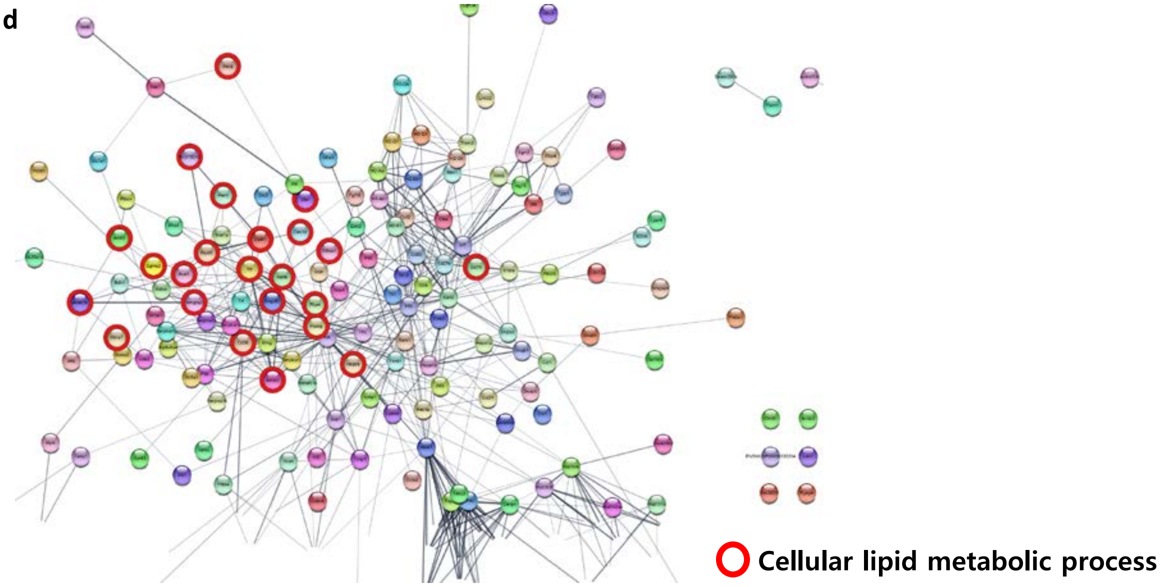

Supplementary Fig. 5: RNA sequencing and systemic analysis of Evo targeting genes. (a) Multidimensional Scaling (MDS) analysis showed that the genetic change patterns of the WT, Con and Evo treatment groups were clearly distinguished by group. (b) Expressing the quantitative difference in gene expression by group as a heat map. (c) Results of enrichment of biological processes (BP) pathways of genes significantly changed by EVO through gene ontology analysis. (d) STRING protein interaction map of genes significantly changed by Evo.

Supplementary References:

1. Bolger, A.M., Lohse, M. & Usadel, B. Trimmomatic: a flexible trimmer for Illumina sequence data. *Bioinformatics* **30**, 2114-2120 (2014).
2. Kim, D., Langmead, B. & Salzberg, S.L. HISAT: a fast spliced aligner with low memory requirements. *Nat. Methods.* **12**, 357-360 (2015).
3. Feng, J. *et al*. GFOLD: a generalized fold change for ranking differentially expressed genes from RNA-seq data. *Bioinformatics* **28**, 2782-8 (2012).
4. Raudvere, U. *et al.* g:Profiler: a web server for functional enrichment analysis and conversions of gene lists (2019 update). *Nucleic Acids Res.* **47**, W191-W8 (2019).
5. Szklarczyk, D. *et al*. The STRING database in 2021: customizable protein-protein networks, and functional characterization of user-uploaded gene/measurement sets. *Nucleic Acids Res.* **49**, D605-D12 (2021).
